# Gate-controlled quantum dots and superconductivity in planar germanium

**DOI:** 10.1038/s41467-018-05299-x

**Published:** 2018-07-19

**Authors:** N. W. Hendrickx, D. P. Franke, A. Sammak, M. Kouwenhoven, D. Sabbagh, L. Yeoh, R. Li, M. L. V. Tagliaferri, M. Virgilio, G. Capellini, G. Scappucci, M. Veldhorst

**Affiliations:** 1 QuTech and Kavli Institute of Nanoscience, Delft University of Technology, PO Box 5046, 2600 GA Delft, The Netherlands; 2 QuTech and the Netherlands Organisation for Applied Scientific Research (TNO), Stieltjesweg 1, 2628 CK Delft, The Netherlands; 3 Dipartimento di Fisica “E. Fermi”, Università di Pisa, Largo Pontecorvo 3, 56127 Pisa, Italy; 4 Dipartimento di Scienze, Università degli studi Roma Tre, Viale Marconi 446, 00146 Roma, Italy; 5 0000 0001 0142 678 IHP, Im Technologiepark 25, 15236 Frankfurt (Oder), Germany

## Abstract

Superconductors and semiconductors are crucial platforms in the field of quantum computing. They can be combined to hybrids, bringing together physical properties that enable the discovery of new emergent phenomena and provide novel strategies for quantum control. The involved semiconductor materials, however, suffer from disorder, hyperfine interactions or lack of planar technology. Here we realise an approach that overcomes these issues altogether and integrate gate-defined quantum dots and superconductivity into germanium heterostructures. In our system, heavy holes with mobilities exceeding 500,000 cm^2^ (Vs)^−1^ are confined in shallow quantum wells that are directly contacted by annealed aluminium leads. We observe proximity-induced superconductivity in the quantum well and demonstrate electric gate-control of the supercurrent. Germanium therefore has great promise for fast and coherent quantum hardware and, being compatible with standard manufacturing, could become a leading material for quantum information processing.

## Abstract (web summary)

Superconductor–semiconductor hybrid systems can bring together physical properties that are promising for fast and coherent quantum technology. Here, Hendrickx et al. realize such a system in planar germanium heterostructures demonstrating excellent quantum dots and tunable Josephson supercurrents.

## Introduction

Solid state quantum computing is actively pursued using superconducting and semiconducting materials^1–3^. The group-IV semiconductors Si and Ge come with central advantages for the realisation of spin quantum bits (qubits). Not only has their purity and technology been refined to a formidable level, they also possess an abundant isotope with zero nuclear spin^4,5^, enabling spin qubits to reach extremely long coherence times^6,7^ and high fidelity^8^. These powerful properties have led to demonstrations of two-qubit logic gates^9,10^ and quantum algorithms^11^. The exchange interaction that is central in these demonstrations is local and cannot directly be used to couple qubits at a distance. Instead, long-range coupling of spin qubits is being explored by incorporating superconductivity and in a first step strong spin–photon coupling has been achieved^12,13^.

Hole quantum dots in Ge are particularly promising in this context. Ge has the highest hole mobility of all known semiconductors^14^, reaching values up to 1,500,000 cm^2^ (Vs)^−1^ in doped heterostructures^15^ and is expected to host strong spin–orbit coupling^16,17^, which facilitates electrical driving for fast qubit operations^18^. Furthermore, the valence band in Ge has no valley degeneracy, so, compared to electrons^19^, hole qubits do not have the complication of these close quantum levels^16^. Experiments have shown readout of holes in Ge/Si nanowires^20,21^, self-assembled quantum dots^22^ and hut wires^23^, and promising spin lifetimes have been found^23,24^. In addition, the strong Fermi-level pinning at the valence band edge leads to ohmic behaviour for all metal–(*p*-type) Ge contacts^25^. The resulting strong coupling between metal and semiconductor enables the fabrication of hybrid devices of quantum dot and superconducting structures^26,27^.

Now, the crucial next step is the development of a planar platform that can bring together low disorder, potential for fast driving and avenue for scaling. Here we address this challenge and present the formation of a quantum dot in a planar Ge quantum well. Furthermore, we implement direct Al-based ohmic contacts that eliminate the need for dopant implantation. In addition, the Al leads can proximity-induce superconductivity in the quantum well and we can control the associated supercurrent by tuning electrical gates.

## Results

### Ge/SiGe heterostructure

**Fig. 1:**
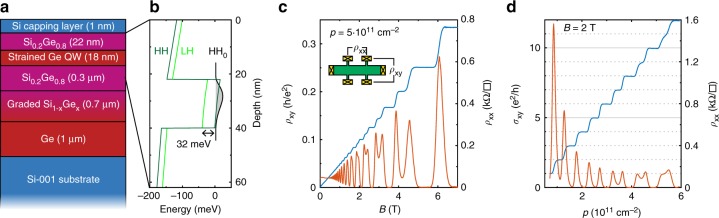
Ge/SiGe heterostructure and magnetotransport measurements. **a** Schematic representation of the full heterostructure (dimensions not to scale). **b** Simulated band structure within the SiGe–Ge–SiGe heterostructure, showing strain-induced splitting of the heavy and light hole band edges and strong confinement of heavy holes in the Ge. **c**, **d** Magnetotransport measurements in a perpendicular magnetic field *B*, showing clear Shubnikov–de Haas oscillations and quantised Hall conductance as a function of *B* (**c**) and the carrier sheet density *p* (**d**) as converted from the top gate potential using low-field Hall effect data

Si and Ge are completely miscible and the lattice constant of their alloy, SiGe, varies continuously between its constituents. This is exploited by using strain-relaxed compositionally graded SiGe layers as virtual substrates to define Ge/SiGe heterostructures as shown schematically in Fig. 1a. We use a high-throughput reduced-pressure chemical vapour deposition (RP-CVD) reactor to grow the complete heterostructure in one deposition cycle on a 100 mm Si(001) substrate. The Ge quantum well is deposited pseudomorphically on a strain-relaxed Si_0.2_Ge_0.8_/Ge/Si virtual substrate obtained by reverse grading^28^ (see Methods). The resulting in-plane compressive strain in the quantum well splits the valence band states, which further increases the hole mobility^29^. The quantum well layer is separated from the surface by a Si_0.2_Ge_0.8_ spacer and a Si cap.

Because of the type-I band alignment in Ge-rich Ge/SiGe heterostructures, the valence band maximum is energetically higher in Ge than in SiGe, such that holes accumulate in the Ge quantum well^30^. Heavy and light hole (HH and LH, respectively) electronic states and the related band profiles have been calculated by solving the Schrödinger–Poisson equation for low temperatures as a function of vertical electric field. In our simulations, both a multi-valley effective mass approach^31^ and an atomistic tight-binding model^32^ have been used, obtaining consistent data. The calculated valence band edge profile is shown in Fig. 1b, where the wave function of the fundamental state HH_0_, which has HH symmetry and is well confined in the Ge layer, is also sketched. The strain-induced splitting of the HH and LH band edges in the Ge region increases the energy of the fundamental LH state LH_0_ (not shown), which is found at about 46 meV above HH_0_. Furthermore, we estimate a separation of 14 meV for the first excited HH level HH_1_ (not shown). Notice that these energy splittings are significantly larger than those resulting from the valley interaction in Si/SiO_2_ and Si/SiGe devices, and thus excellent conditions for the operation of spin qubits are expected in this material.

The hole mobility is measured in magnetotransport experiments at 50 mK using a heterostructure field effect transistor as shown in the inset of Fig. 1c. Here the yellow boxes indicate metallic ohmic contacts to the quantum well created by diffusion into the top SiGe layer and the green structure represents an isolated Hall-bar-shaped top gate, which is used to control the hole density in the quantum well. Clear Shubnikov–de Haas oscillations with zero-resistivity minima and Zeeman splitting of the Landau levels at higher fields are observed in the longitudinal resistance *ρ*_*xx*_ as a function of the magnetic field *B* (Fig. 1c). Furthermore, we observe flat quantum Hall effect (QHE) plateaus at values 1/*ν* for integer *ν* in the transverse resistance *ρ*_*xy*_ in units of *h*/*e*^2^, where *h* is Planck’s constant and *e* the elementary charge. The investigated heterostructures support a density of up to 6 × 10^11^ cm^−2^ and have a maximum mobility of more than 500,000 cm^2^ (Vs)^−1^, corresponding to a mean free path of *L*_m_ = 6.4 μm and providing new benchmarks for holes in undoped structures. Figure 1d shows the Hall conductivity *σ*_*xy*_ in units of *e*^2^/*h* as a function of the hole density *p*, controlled by the top gate potential. Again, zero-resistivity minima in *ρ*_*xx*_ and clear linear quantisation steps in *σ*_*xy*_ are observed. This demonstrates the control of the hole density over a large range using the top gate, which is a central prerequisite for the formation of electrostatically defined quantum dots.

### Quantum dot

**Fig. 2:**
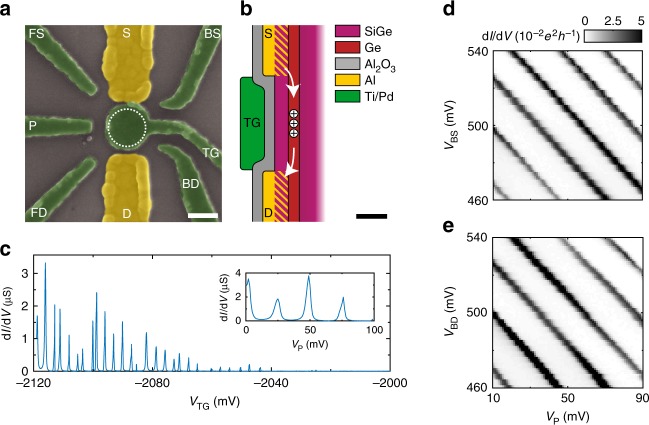
Fabrication and operation of a gate-defined quantum dot. **a** False-coloured SEM image of the quantum dot device. The quantum dot is defined under the top gate TG (dotted circle) and its occupancy can be controlled by the central plunger gate P. BS and BD correspond to source and drain barriers, respectively; FS and FD are finger gates for additional control. Scale bar is 100 nm. **b** Schematic of the device gate layers, showing the top gate and the ohmic contacts achieved by in-diffusion of Al. Scale bar is 50 nm. **c** Transport measurements showing Coulomb oscillations as a function of the top gate with *V*_P_ = 0 mV and as a function of the plunger gate with *V*_TG_ = −2120 mV (inset). **d**, **e** Influence of the barrier gates BS with *V*_*BD*_ = 500 mV (**d**) and BD with *V*_*BS*_ = 500 mV (**e**) on the observed conductivity. The coupling of the two individual gates to the quantum dot is nearly identical, emphasising the excellent homogeneity reached in this system

A scanning electron microscope (SEM) image of the quantum dot nanostructure is shown in Fig. 2a. Here the ohmic Al contacts are coloured in yellow and the isolated Ti/Pd gates are shown in green. In a first step, the Al contacts to the Ge quantum well are defined by electron beam lithography, local etching of the Si capping layer and thermal evaporation of Al. Subsequently, an Al_2_O_3_ gate dielectric is grown by atomic layer deposition at 300 °C, which also serves as an annealing step to enable the diffusion of Al into the SiGe spacer. In the Ti/Pd gate layer, we design a circular top gate between the two Al leads under which a single quantum dot will be formed. In addition, a central plunger gate P is included to control the dot occupation, as well as barrier gates (BS and BD) and additional finger gates (FS and FD) in the corners of the device. These allow for additional control of the dot size and the tunnelling rates between the quantum dot and the source and drain leads.

A conceptual drawing of the device cross-section is shown in Fig. 2b, where the diffused Al leads that contact the Ge quantum well are indicated by the stripe-patterned regions. Because of the ohmic nature of the contact, the transport through the quantum dot can be measured without the need for additional reservoir gates and dopant implantation. As a result, no annealing step at temperatures higher than the quantum well deposition temperature (500 °C) is needed during the fabrication, avoiding harmful Ge/Si intermixing at the interface^29^.

When measuring the source drain conductance d*I*/d*V* as a function of the top gate voltage, conductance peaks are expected when the dot potential aligns between the source and drain potentials, which are the so-called Coulomb oscillations. This is shown in Fig. 2c, where d*I*/d*V* was measured as a function of the top gate voltage *V*_TG_. The expected oscillations are observed, which is a clear sign of the formation of a quantum dot. The spacing between the peaks is quite regular, indicating that the quantum dot is operated in the many-hole regime. From the period of the conductance peaks, we can extract a top gate capacitance of ~56 aF, which is in very good agreement with the expected capacitance of 52 aF of a parallel plate capacitor using the lithographic dimensions of the top gate. When the top gate voltage is increased and the quantum dot is depleted, the amplitude of the observed peaks is reduced and eventually vanishes, as the tunnelling rates to source and drain reservoirs drop as an effect of the reduced size of the dot. As shown in the supplementary material, we are able to circumvent this effect using an additional accumulation gate in a different device, which allows us to reach the few-hole regime (Supplementary Fig. 1). When TG is tuned to the quantum dot regime, similar oscillations are observed as a function of the plunger gate voltage *V*_P_. Here a larger spacing of the Coulomb peaks is observed, corresponding to a gate capacitance of ~6.4 aF, in agreement with the expected weaker coupling of P to the quantum dot. Note that because of the structure of the source and drain leads no additional tuning of the device is necessary. Equivalent to a classical transistor, the quantum dot can be defined using a single gate (TG), which bodes well for the scaling up of qubits in this system.

As shown in Fig. 2d, e, the dot occupancy can also be controlled using the barrier gates BS and BD. The observed conductance lines are diagonal and very equivalent for each of the two barriers and the plunger, indicating that the coupling to the quantum dot is nearly identical. This confirms that the quantum dot is formed in a central position under TG and that, as an effect of low disorder in the heterostructure, a very high level of control is achieved. Furthermore, by implementing a second layer of Ti/Pd gates we can also independently control the tunnelling rate between the quantum dot and the reservoir (Supplementary Fig. 2).

**Fig. 3:**
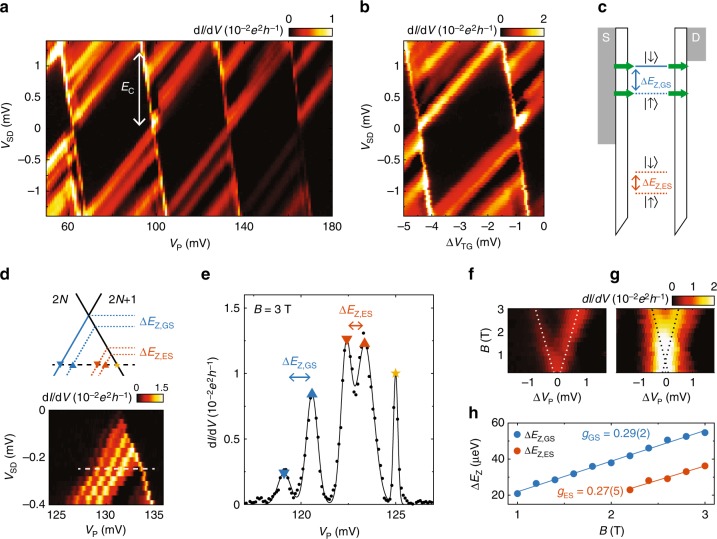
Bias spectroscopy and *g*-factor measurements. **a**, **b** Colour plots of bias spectroscopy as a function of *V*_P_ (**a**) and Δ*V*_TG_ = *V*_TG_ + 2084 mV (**b**), showing Coulomb diamonds with *E*_*C*_ = 1.3(1) meV. **c** Schematic drawing of the Zeeman splitting Δ*E*_Z_ = *gμ*_B_*B* of the quantum dot levels, illustrating the hole transport via the Zeeman-split energy levels. **d** Bias spectroscopy showing the line splitting in a 2.9 T in-plane field. Dashed line indicates the cut plotted in **e**. **e** Differential conductance as a function of Δ*V*_P_ for *B* = 3 T, at *V*_*SD*_ = −0.26 mV. Solid line corresponds to a fit to the data using the sum of five Gaussian profiles. **f**, **g** Differential conductance for both the ground (**f**) and the excited state (**g**) as a function of *B* and Δ*V*_P_, with ${\mathrm{\Delta }}V_{\mathrm{P}} = V_{\mathrm{P}} - \overline V _{{\mathrm{peak}}}$ and $\overline V _{{\mathrm{peak}}}$ being the average voltage of the two transition peaks corresponding to either the ground or the excited state (for raw data, see Supplementary Fig. 3). Dotted lines represent linear fits to the peak positions (same as in **g**). **h** Energy splittings for the ground and excited state as a function of *B*. Solid lines are linear fits to the data yielding the corresponding *g*-factors

To further characterise the quantum dot, we measure d*I*/d*V* as a function of *V*_P_ and the DC bias voltage *V*_SD_. As shown in Fig. 3a, Coulomb diamonds are observed^33^. From the height and width of these diamonds, the charging energy *E*_C_ and the lever arm of the corresponding gate *α* can be extracted. In the regime shown here (*V*_BS_ = *V*_BD_ = 550 mV and *V*_FD_ = 600 mV), we find *α*_P_ = 0.037(3) and *E*_C_ = 1.3(1) meV.

Similar diamonds are observed as a function of *V*_TG_ (Fig. 3b). Here the lever arm is found to be significantly larger (*α*_TG_ = 0.41(3)), as is expected because the dot is formed directly under TG. A substructure is clearly visible in the conducting areas. These additional lines could correspond to either charge transport via excited states of the dot or to a modulation of the density of states within the source and drain reservoirs^34^.

When an external magnetic field *B* is applied, the energy levels of spin degenerate states are expected to split as a result of the Zeeman effect^33^ (cf. Fig. 3c). This becomes apparent as a splitting of the conductance lines related to odd hole occupations 2*N* + 1 of the quantum dot, as shown in Fig. 3d in an in-plane magnetic field *B* = 2.9 T. Both the ground state and the excited state are subject to this splitting, which we extract by fitting the observed conductance for *V*_SD_ = −0.26 mV using Gaussian profiles and multiplying the splitting in voltage with the measured lever arm *α*_P_ (Fig. 3e).

A linear trend is observed as a function of the applied magnetic field as shown in Fig. 3f, g. Note that small splittings Δ*E*_Z_ < 20 μeV could not be resolved because of the finite width of the conductance peaks. We find the effective *g*-factors *g*_GS_ = 0.29(2) and *g*_ES_ = 0.27(5) for the ground state and excited state, respectively, from the linear fits in Fig. 3h. For the excited state, our data point to either a non-linearity at lower fields or a significant zero-field spin splitting Δ*E*_Z,0_ ≈ −11 μ*e*V. The *g*-factor of the pure HH state is expected to vanish completely for an in-plane field. However, the additional confinement of the holes in the *x*,*y*-plane leads to a significant admixture of LH states and a non-zero in-plane Zeeman splitting^35^.

The observed spin splitting of the first line parallel to the diamond edge ground state identifies it as belonging to the first excited state rather than being connected to the reservoir. The measured energy splitting with respect to the ground state Δ*E* ≈ 100 μeV remains unchanged as a function of magnetic field strength. It can be compared to the expected level splitting for a two-dimensional (2D) quantum dot with area *A* and effective hole mass m^*^, which is given by Δ*E* = *π*ℏ^2^/m^*^A. From the temperature dependence of the Shubnikov–de Haas oscillations measured for the Hall bar structures, we find m^*^ = 0.08 *m*_e_, with the electron mass *m*_e_, and with our device geometry (*A* =  0.019 μm^2^) we obtain Δ*E* ≈ 150 μeV, in good agreement with the measured value.

**Fig. 4:**
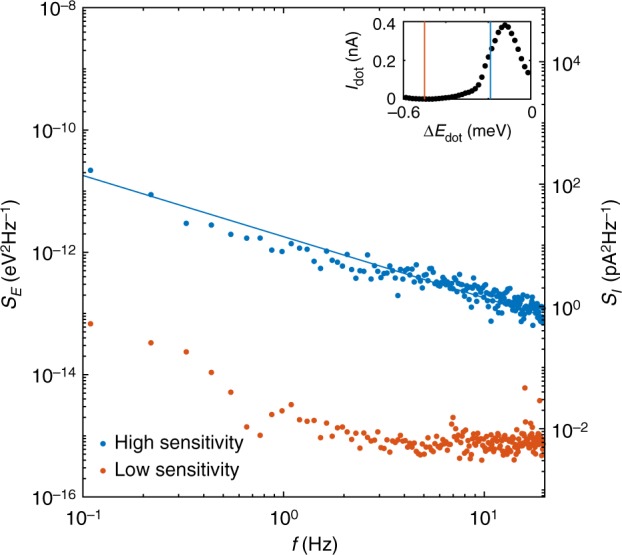
Quantum dot charge noise spectrum. Current noise power spectrum for the quantum dot in transport (blue dots) and in Coulomb blockade (orange dots) as indicated in the inset. Solid line corresponds to an apparent linear fit to the data, yielding a slope of −1.00(4)

The creation of ohmic leads by the diffusion of Al in the direct vicinity of the quantum dot could be suspected of creating additional charge traps that have a negative influence on the coherence of a potential qubit. To quantify this effect, we measure the charge noise acting on our quantum dot by recording the transport current *I*_dot_ in a sensitive region, i.e. on the slope of a Coulomb peak. A 100-s-long time trace of *I*_dot_ is acquired at a sampling rate of 30.5 kHz and is decomposed into 15 traces of equal lengths. The discrete Fourier spectra obtained from these traces are averaged, yielding the noise spectral density *S*_*E*_ presented in Fig. 4 in comparison to the corresponding spectrum measured in a low sensitivity region (Coulomb blockade). The difference between the two traces confirms that the measured low-frequency noise spectrum is indeed dominated by charge noise acting on the quantum dot. The noise spectrum follows a typical 1/*f* trend for low frequencies^36,37^ (solid line in Fig. 4). We find the equivalent detuning noise at 1 Hz to be 1.4 μeV/$\sqrt {{\mathrm{Hz}}}$. This compares well to noise figures at 1 Hz in other materials, such as 7.5, 0.5, or 2.0 μeV/$\sqrt {{\mathrm{Hz}}}$ in GaAs^36^, Si/SiO_2_^37^ and Si/SiGe^37^, respectively, showing the suitability of our approach for the creation of low-noise qubits.

### Josephson field effect transistor (JoFET)

**Fig. 5:**
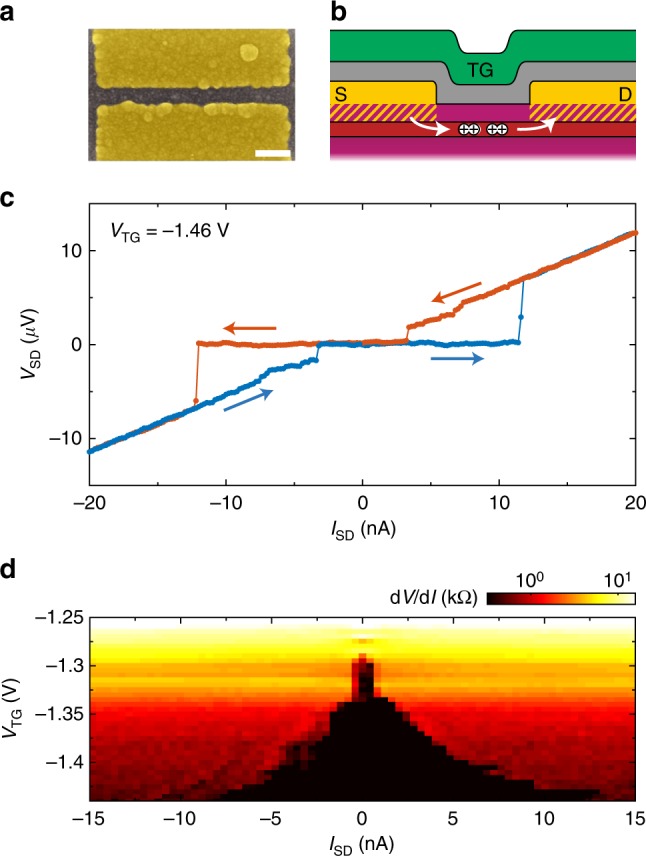
Tunable induced superconductivity in the Ge quantum well. **a** SEM image of the JoFET device with a gap of 100 nm. Scale bar is 200 nm. **b** Device schematic using the colour scheme of Fig. 2b. Superconducting transport through the quantum well is illustrated. **c** Source-drain voltage as a function of the applied current at a top gate voltage of *V*_TG_ = −1.46 V. A flat plateau with zero resistance is observed up to a critical current of *I*_c_ ≈ 12 nA as a clear signature of a supercurrent through the device. **d** The critical current can be modulated by tuning the top gate voltage, as shown in the colour plot of the current dependence of the device resistance as a function of *V*_TG_, where *I*_SD_ was swept from negative to positive values

The direct ohmic contact of the Al leads to the Ge quantum well can proximity-induce superconductivity in the semiconductor system. To demonstrate this effect, we fabricate a JoFET device with two thicker (*d* = 30 nm) and broader (*w* = 1 μm) aluminium leads. These are positioned with a separation of 100 nm and are overlaid with a single top gate; a SEM image of the device is shown together with a schematic drawing of the layer structure in Fig. 5a, b. When the source-drain voltage is measured as a function of the sourced current *I*_SD_ at negative top gate potential, a zero-resistance plateau is observed as a clear sign of a supercurrent. This is shown in Fig. 5c, where the blue and orange traces represent measurements in different sweep directions, respectively. Sweeping towards higher absolute values of *I*_SD_, the critical current *I*_c_ is reached at ~12 nA. Beyond these values, linear (ohmic) behaviour is observed. A hysteresis can be observed when sweeping back, which can be expected for proximity induced SNS-junctions and is usually caused by self-heating of the junction when it exits the superconducting state^38^. To demonstrate that this supercurrent is induced in the quantum well, we measure the observed bias current dependence of the source-drain resistance d*V*/d*I* as a function of the top gate potential *V*_TG_, shown in Fig. 5d. The critical current, which defines the borders of the zero resistance range observed as a black region in the colour plot, is reduced towards increasing *V*_TG_. This is an effect of the decrease in the carrier density and the resulting increase of the normal state resistance. For voltages above *V*_TG_ ≈ −1.27 V, *I*_c_ cannot be resolved at the limited instrumental resolution in our measurements. For decreasing gate potential, we find that the increase in *I*_c_ is more significant than the reduction in the normal resistance *R*_n_. The characteristic voltage *I*_c_*R*_n_ hence increases and reaches values higher than ~10 μV. Additionally, a modulation of the critical current can be observed as a function of magnetic field, which is a clear hallmark of the Josephson effect (Supplementary Fig. 4). These proof-of-principle experiments should be followed by a detailed characterisation of the proximity-induced superconductivity in this platform. Interesting future paths of research include the study of ballistic transport and the investigation and optimisation of the sharpness of the Al–Ge interface, which could significantly increase the *I*_c_*R*_n_ product.

## Discussion

In conclusion, we have shown the operation of a hole quantum dot in a planar, undoped and buried Ge quantum well with a record hole mobility. The Al ohmic leads to the quantum dot significantly simplify the fabrication and tuning processes without an increase of the measured charge noise in comparison to other systems. The strong capacitive coupling between the superconductor and the quantum dot makes this system ideal for reaching strong spin–photon coupling, while the strong spin–orbit coupling present in HHs could be exploited for fast electrical qubit driving. Furthermore, the demonstration of gate-tunable superconductivity opens up new research directions, including Majorana modes^39^ and gatemons^27^. Hole quantum dots in planar Ge constitute thereby a versatile platform that can leverage semiconductor manufacturing to advance and broaden the field of quantum computing.

## Methods

### Heterostructure growth

The Ge/SiGe heterostructures were grown in one deposition cycle in an Epsilon 2000 (ASMI) RP-CVD reactor on a 100 mm *n*-type Si(001) substrate (resistivity 5 Ω cm). The growth starts with a 1-μm-thick layer of Ge, using a dual-step process with initial low-temperature (400 °C) growth of a Ge seed layer followed by a higher-temperature (625 °C) overgrowth of a thick relaxed Ge buffer layer. A cycle anneal at 800 °C is performed to promote full relaxation of the Ge. Subsequently, a 700 nm reverse-graded Si_1−*x*_Ge_*x*_ layer is grown at 800 °C with *x* changing linearly from 1 to 0.8. A relaxed 300 nm buffer Si_0.2_Ge_0.8_ is then grown in two steps at an initial high temperature of 800 °C followed by a low-temperature growth at 500 °C. The growth continues with a 18-nm-thick strained Ge quantum well, a 22-nm-thick Si_0.2_Ge_0.8_ barrier and a 1-nm-thick Si cap. The final layers are all deposited at a temperature of 500 °C.

### Device fabrication

The photolithography process for fabricating the Hall bar field effect transistors includes the following steps: mesa etching, deposition of 60-nm-thick Pt layer ohmic contacts, and atomic layer deposition (ALD) at 300 °C of a 30-nm-thick Al_2_O_3_ dielectric to isolate a Ti/Au top gate (thicknesses 10/150 nm).

For the quantum dot, the contact and gate structures are created by electron beam lithography, electron beam evaporation of Al and Ti/Pd and lift-off. Following the Al contact layer (20 nm), ALD is used to grow 17 nm of Al_2_O_3_ at 300 °C as a gate dielectric, followed by the Ti/Pd (5/35 nm) gate structures. For the JoFET device, the same process is done with layer thicknesses 30 nm (Al), 25 nm (Al_2_O_3_) and 5/35 nm (Ti/Pd).

### Measurement system

Magnetotransport data has been obtained in a ^3^He dilution refrigerator with a base temperature of 50 mK, equipped with a 9 T magnet. The carrier density $p = \left( {\left| e \right|\frac{{\mathrm{d}\rho _{yx}}}{{\mathrm{d}B}}} \right)^{ - 1}$ in the quantum well is derived from the measured Hall resistivity for fields *B* < 0.3 T, where no QHE steps are observed. This density can also be derived from the Fourier transform of the longitudinal resistance data as a function of 1/*B*. From the observed peak width of 1.5 T, we estimate an upper bound for the zero-field spin splitting in our two-dimensional hole gas of 1.5 meV. All quantum dot and JoFET measurements were performed in a ^3^He dilution refrigerator with a base temperature of <10 mK, equipped with a 3 T magnet. All quantum dot measurements of d*I*/d*V* are performed using lock-in amplification with typical modulation amplitude and frequency of *δV*_SD_ = 10–100 μV and *f*_mod_ = 73.5 Hz, respectively, and a small offset in bias voltage due to the measurement electronics is subtracted. The JoFET device was measured in a four-point configuration, sourcing a current and measuring the potential across the superconducting junctions. The plotted voltage is corrected by a small offset of the measurement electronics. The differential resistance is measured using lock-in amplification with typical a modulation amplitude of *δI*_SD_ = 0.3 nA.

### Data availability

All data underlying this study are available from the 4TU ResearchData repository at 10.4121/uuid:6a4c90c9-1fd7-442f-bc21-8fbe6876e1ea.

## Electronic supplementary material

Supplementary Information

## References

[1] Nakamura Y, Pashkin YA, Tsai JS (1999). Coherent control of macroscopic quantum states in a single-Cooper-pair box. Nature.

[2] Petta JR (2005). Coherent manipulation of coupled electron spins in semiconductor quantum dots. Science.

[3] Basov DN, Averitt RD, Hsieh D (2017). Towards properties on demand in quantum materials. Nat. Mater..

[4] Itoh K (1993). High purity isotopically enriched 70-Ge and 74-Ge single crystals: Isotope separation, growth, and properties. J. Mater. Res..

[5] Itoh KM, Watanabe H (2014). Isotope engineering of silicon and diamond for quantum computing and sensing applications. MRS Commun..

[6] Veldhorst M (2014). An addressable quantum dot qubit with fault-tolerant control-fidelity. Nat. Nanotechnol..

[7] Sigillito A (2015). Electron spin coherence of shallow donors in natural and isotopically enriched germanium. Phys. Rev. Lett..

[8] Yoneda J (2017). A quantum-dot spin qubit with coherence limited by charge noise and fidelity higher than 99.9. Nat. Nanotechnol.

[9] Veldhorst M (2015). A two-qubit logic gate in silicon. Nature.

[10] Zajac DM (2018). Resonantly driven CNOT gate for electron spins. Science.

[11] Watson TF (2018). A programmable two-qubit quantum processor in silicon. Nature.

[12] Mi X (2018). A coherent spin–photon interface in silicon. Nature.

[13] Samkharadze N (2018). Strong spin-photon coupling in silicon. Science.

[14] Pillarisetty R (2011). Academic and industry research progress in germanium nanodevices. Nature.

[15] Failla M (2016). Terahertz quantum Hall effect for spin-split heavy-hole gases in strained Ge quantum wells. New J. Phys..

[16] Terrazos, L. A. et al. Light-mass hole-spin qubits formed in a Ge quantum well. Preprint at http://arxiv.org/abs/1803.10320 (2018).

[17] Moriya R (2014). Cubic Rashba spin-orbit interaction of a two-dimensional hole gas in a strained-Ge-SiGe quantum well. Phys. Rev. Lett..

[18] Watzinger, H. et al. Ge hole spin qubit. Preprint at http://arxiv.org/abs/1802.00395 (2018).

[19] Yang CH (2012). Orbital and valley state spectra of a few-electron silicon quantum dot. Phys. Rev. B.

[20] Lu W, Xiang J, Timko BP, Wu Y, Lieber CM (2005). One-dimensional hole gas in germanium/silicon nanowire heterostructures. PNAS.

[21] Hu Y (2007). A Ge/Si heterostructure nanowire-based double quantum dot with integrated charge sensor. Nat. Nanotechnol..

[22] Ares N (2013). Nature of tunable hole g factors in quantum dots. Phys. Rev. Lett..

[23] Vukušić, L. et al. Single-shot readout of hole spins in Ge. Preprint at http://arxiv.org/abs/1803.01775 (2018). 10.1021/acs.nanolett.8b03217 PMC6243395 30359041

[24] Hu Y, Kuemmeth F, Lieber CM, Marcus CM (2012). Hole spin relaxation in Ge–Si core–shell nanowire qubits. Nat. Nanotechnol..

[25] Dimoulas A, Tsipas P, Sotiropoulos A, Evangelou EK (2006). Fermi-level pinning and charge neutrality level in germanium. Appl. Phys. Lett..

[26] Katsaros G (2010). Hybrid superconductor–semiconductor devices made from self-assembled SiGe nanocrystals on silicon. Nat. Nanotech..

[27] Larsen T (2015). Semiconductor-nanowire-based superconducting qubit. Phys. Rev. Lett..

[28] Shah VA (2008). Reverse graded relaxed buffers for high Ge content SiGe virtual substrates. Appl. Phys. Lett..

[29] Dobbie A (2012). Ultra-high hole mobility exceeding one million in a strained germanium quantum well. Appl. Phys. Lett..

[30] Virgilio M, Grosso G (2006). Type-I alignment and direct fundamental gap in SiGe based heterostructures. J. Phys. Condens. Matter.

[31] Virgilio M (2014). Physical mechanisms of intersubband-absorption linewidth broadening in s-Ge/SiGe quantum wells. Phys. Rev. B.

[32] Busby Y (2010). Near- and far-infrared absorption and electronic structure of Ge-SiGe multiple quantum wells. Phys. Rev. B.

[33] Hanson R, Kouwenhoven LP, Petta JR, Tarucha S, Vandersypen LMK (2007). Spins in few-electron quantum dots. Rev. Mod. Phys..

[34] Escott CC, Zwanenburg FA, Morello A (2010). Resonant tunnelling features in quantum dots. Nanotech.

[35] Nenashev AV, Dvurechenskii AV, Zinovieva AF (2003). Wave functions and g factor of holes in Ge/Si quantum dots. Phys. Rev. B.

[36] Basset J (2014). Evaluating charge noise acting on semiconductor quantum dots in the circuit quantum electrodynamics architecture. Appl. Phys. Lett..

[37] Freeman BM, Schoenfield JS, Jiang H (2016). Comparison of low frequency charge noise in identically patterned Si/SiO2 and Si/SiGe quantum dots. Appl. Phys. Lett..

[38] Courtois H, Meschke M, Peltonen JT, Pekola JP (2008). Origin of hysteresis in a proximity Josephson junction. Phys. Rev. Lett..

[39] Mourik V (2012). Signatures of Majorana fermions in hybrid superconductor-semiconductor nanowire devices. Science.

