# Supplementary material for: Gate-controlled quantum dots and superconductivity in planar germanium
Source: Nat Commun. 2018 Jul 19;9:2835. doi: 10.1038/s41467-018-05299-x (PMC6053419; doi:10.1038/s41467-018-05299-x)
Supplement: Supplementary file 1 — Supplementary Information [file 41467_2018_5299_MOESM1_ESM.pdf]

Supplementary Information: Gate-controlled quantum dots and superconductivity in planar germanium

Hendrickx et al.

(Dated: June 15, 2018)

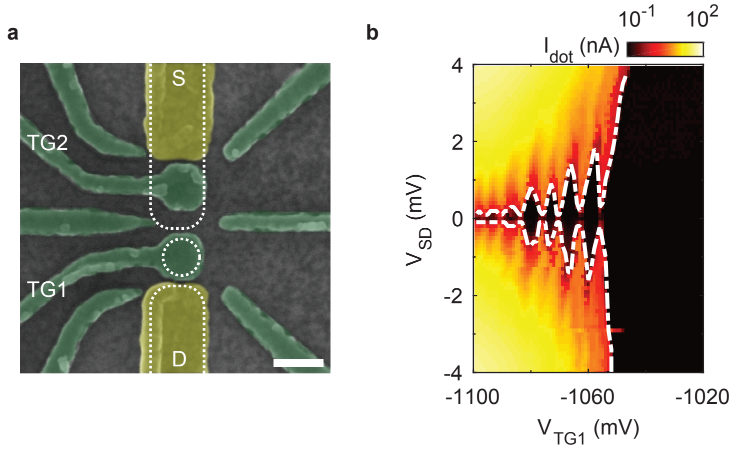

Supplementary Figure 1. Device operating in the few-hole regime a, False-coloured SEM image of device 2 with topgates TG1 and TG2. Al, ohmic leads are coloured in yellow and Ti/Pd gates are depicted in green. Dashed lines indicates the accumulation of holes and illustrate the extended reservoir under gate TG2, together with the QD under gate TG1. Scale bar is 100 nm. **b**, Bias spectroscopy of device 2. The device is measured in a single dot operation mode where TG2 is set to high negative voltage ($V_{\mathrm{TG2}} = -2500$ mV) and thus acts as an extended source reservoir. The additional barrier and finger gates are set to $V \sim 1000$ mV. Dashed contour lines correspond to $I_{\mathrm{SD}} = 0.2$ nA and show a clear increase of E_C with lower occupation, indicating the device is operated in the few-hole regime.

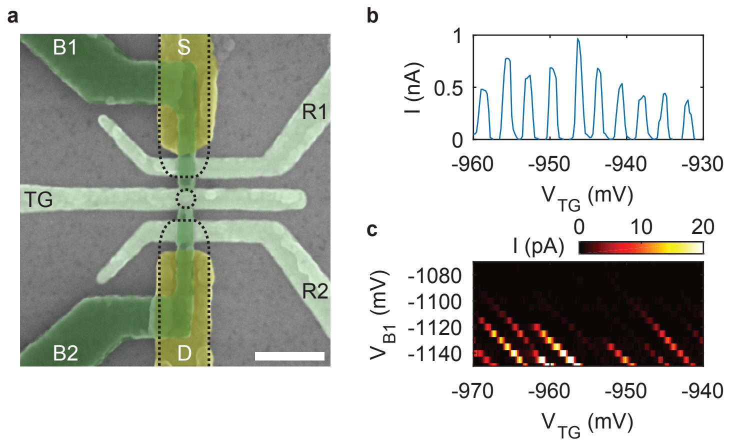

Supplementary Figure 2. Device with independent barrier control a, False-coloured SEM image of device 3, consisting of Al ohmic leads (yellow), a first layer of Ti/Pd gates (light-green) and a second layer of overlapping Ti/Pd gates (dark-green). The ohmics and the two gate layers are separated by respectively 7 and 10 nm of Al_2O_3. Dashed lines indicate the accumulation of holes, indicating the extended reservoirs underneath gates R1 and R2, together with the QD under gate TG. Scale bar is 200 nm. **b**, Transport measurement showing Coulomb oscillations for device 3, as a function of the top gate TG, indicating the formation of a quantum dot. **c**, Colour plot of the transport current as a function of gates B1 and TG. The conduction peaks are observed to fade out for increasing V_{B1}, indicating an independent tunnelling rate control between the quantum dot and the reservoir underneath gate R1.

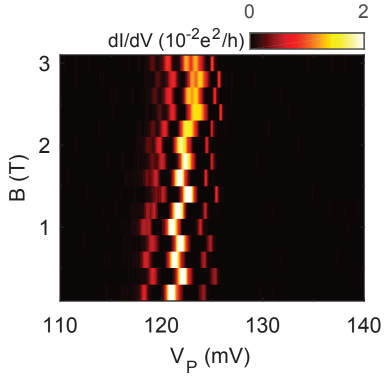

Supplementary Figure 3. **Unprocessed data from figure 3f,g.** Colour plot of dI/dV as a function of plunger gate voltage V_P and in-plane magnetic field B. The sweeping direction is reversed any other line and a weak hysteresis becomes apparent, as well as a slow drift of the line position with time.

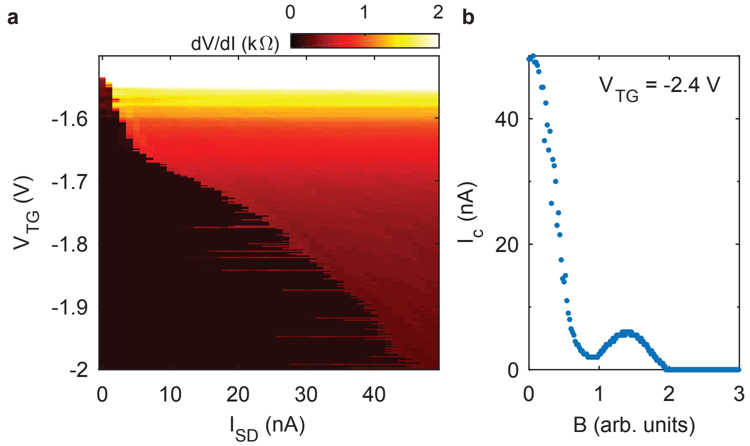

Supplementary Figure 4. **Additional JoFET measurements a**, Colour plot of the differential resistance of a 150-nm-long junction (width w = 1 μm) as a function of the top gate voltage V_{TG}, again showing a gate-tunable supercurrent, with critical currents observed up to 45 nA. **b**, A modulation of the critical current as a function of the applied magnetic field B can be observed, as a clear sign of a Josephson supercurrent. Above $B > 2$, the critical current is smaller than our detection limit and cannot be resolved.

SUPPLEMENTARY METHODS

To reduce detrimental fields and increase the resolution of the magnetic field we can apply, the JoFET device was mounted outside the core of the superconducting magnet. While the remaining stray field allowed for measuring the magnetic field dependence of I_C, the exact magnitude of B has not been calibrated and can hence only be estimated. From the expected stray field, we estimate B to be on the order of a few mT, in agreement with the expected flux for one flux quantum in the junction with area $A = 0.1\ \mu\mathrm{m}^2$.
